# Silica Nanobottles Filled with Photo-cross-linked GelMA for the Sustained Release of Hydrophilic Biomacromolecules

**DOI:** 10.1021/acs.nanolett.5c04288

**Published:** 2025-11-24

**Authors:** Dong Zhang, Yuxuan Meng, Younan Xia

**Affiliations:** † The Wallace H. Coulter Department of Biomedical Engineering, 1372 Georgia Institute of Technology and Emory University, Atlanta, Georgia 30332, United States; § School of Chemistry and Biochemistry, Georgia Institute of Technology, Atlanta, Georgia 30332, United States

**Keywords:** controlled release, drug delivery, nanobottle, cross-linking, hydrophilic payload

## Abstract

Nanobottles have garnered attention as a class of advanced carriers for drug delivery, because of their advantages, such as convenient loading, high capacity, and a well-defined wall for surface modification. However, strategies to achieve sustained release of hydrophilic biomolecules remain limited. Here, we report a hybrid delivery platform that integrates silica nanobottles with photo-cross-linked gelatin methacryloyl for the encapsulation and controlled release of proteins, enzymes, and polysaccharides. Upon cross-linking, the payloads show enhanced intracellular stability, with minimal release in A549 cells for up to 24 h. This system also enables control over release kinetics, with the half-life extendible to several weeks depending on the type and/or molecular weight of the payload. Importantly, released biomacromolecules retain structural and functional integrity, and delivery of nerve growth factor promotes robust neurite outgrowth from PC12 cells. This hybrid system provides a versatile and programmable platform for the long-term delivery of diverse hydrophilic therapeutics.

## Graphical abstract

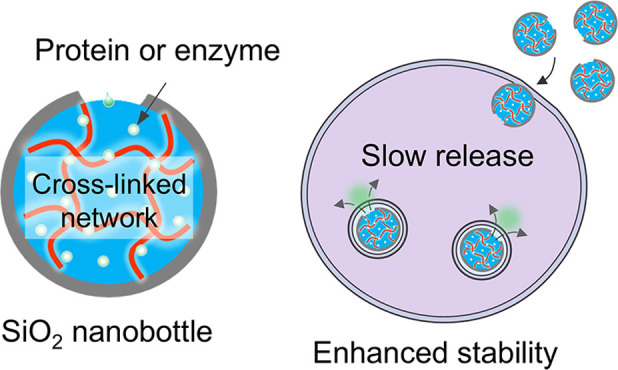

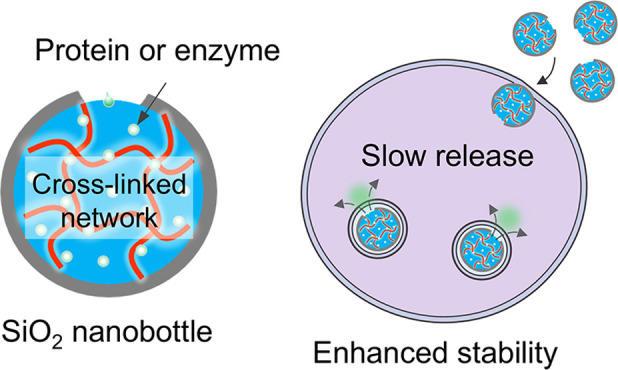

Nanobottles are colloidal particles bearing a hollow interior and a single, controllable opening in the wall.^1,2^ They can be fabricated from polymers, ceramics, metals, and composites, making them highly versatile for different applications.^3–5^ Typical methods for generating nanobottles include conformal coating followed by programmed calcination,^6^ site-selected growth followed by etching,^7^ solvent extraction or extrusion,^8^ swelling followed by freeze-drying,^9^ and swelling-induced symmetry breaking.^10–12^ Among them, the last method seems to be most practical, because of its simplicity and scalability, as well as the capability to produce structures with tunable morphologies. In this approach, solvent swelling provides the driving force to punch an opening in the shell precoated on the bead while creating a cavity.^13–15^ Typically, the surface of a non-cross-linked polystyrene (PS) bead is coated with a thin shell made of silica,^10–12^ polydopamine,^15^ or polyelectrolyte,^16^ followed by exposure to a good solvent for PS in an aqueous solution or emulsion. As the PS matrix is swollen by the solvent, the increased internal pressure will induce protrusion from the weakest site on the shell for the creation of a Janus particle. A nanobottle is then obtained by dissolving all the PS with the same solvent.

When serving as a carrier, nanobottles offer immediate advantages for controlled release applications. Their hollow interior offers a high loading capacity with minimal carrier material,^17^ while the single opening enables efficient loading, discharge, and control of release kinetics across a wide range from rapid delivery to sustained release. The opening can also be sealed with a cap or cork to protect encapsulated payloads from evaporation, contamination, or degradation, a feature crucial for sensitive bioactive molecules.^18^ Moreover, their geometric shape and surface properties can be engineered to optimize interactions with biological systems such as cells and tissues.^19^ Payload release from nanobottles is generally regulated by three strategies.^9,20,21^ The first strategy controls the opening size to modulate the diffusion kinetics of molecular species. The second approach uses a stimuli-responsive cap as an “on–off” gate to maneuver the release. The third method coloads the drug with a solid matrix material (e.g., a phase-change material) to regulate the diffusion rate of the payload. These strategies can also be combined to leverage the merits of two or even all three strategies for the achievement of a tight and customizable control over the release profile. However, the first two methods often result in rapid release (seconds to hours), limiting their use in long-term delivery. The matrix-assisted strategy is more suitable for sustained release, but prior work has relied mainly on hydrophobic fatty acids, which are incompatible with hydrophilic biomolecules such as proteins and enzymes.

Different from previously reported controlled-release systems, here we describe a hybrid platform that integrates nanobottles with a hydrophilic, cross-linkable matrix. SiO_2_ nanobottles were fabricated via swelling-induced symmetry breaking and loaded with a hydrophilic payload together with gelatin methacryloyl (GelMA) precursor. Upon photo-cross-linking, GelMA forms a three-dimensional network that slows payload diffusion, enabling controllable and sustained release. Recent progress in hydrogel-based delivery systems, particularly those employing protein-derived materials such as gelatin, collagen, and zein, have advanced our capability to control the delivery of therapeutics.^22–25^ Building on these advances, our nanobottle-GelMA hybrid platform achieves burst-free release with a tunable half-life from several days to weeks, depending on payload molecular weight.

Figure 1A illustrates the fabrication procedure: the commercial PS beads were coated with a ca. 15 nm SiO_2_ shell and then swollen in aqueous tetrahydrofuran (THF)/sodium dodecyl sulfate (SDS) to generate PS-SiO_2_ Janus particles.^10–12^ We then dissolved all the PS using pure THF for the generation of a SiO_2_ nanobottle characterized by a uniform diameter of ca. 530 nm and a surface opening of ca. 120 nm (Figures 1B–D). Statistical analysis of transmission electron microscopy (TEM) images showed that over 75% of the nanobottles had openings between 100 and 130 nm, demonstrating a narrow size distribution suitable for controlled release. The overall yield, defined as the fraction of structurally intact nanobottles among all particles observed under TEM, was approximately 65%–70%. A representative TEM image highlighting defective structures is shown in Figure S1. The nanobottles were then loaded with fluorescein isothiocyanate-bovine serum albumin (FITC-BSA) (Mw ≈ 66 kDa), GelMA precursor, and Irgacure 2959 at 37 °C, followed by washing and redispersion in cold (4 °C) water. The presence of some solids inside the SiO_2_ nanobottles indicated the successful loading of FITC-BSA into their cavity (Figure 1D). The spatial overlap between the green dots in the fluorescence micrograph and the particles observed in the bright-field image indicates that FITC-BSA was indeed encapsulated in the nanobottles (Figure 1E).

**Figure 1:**
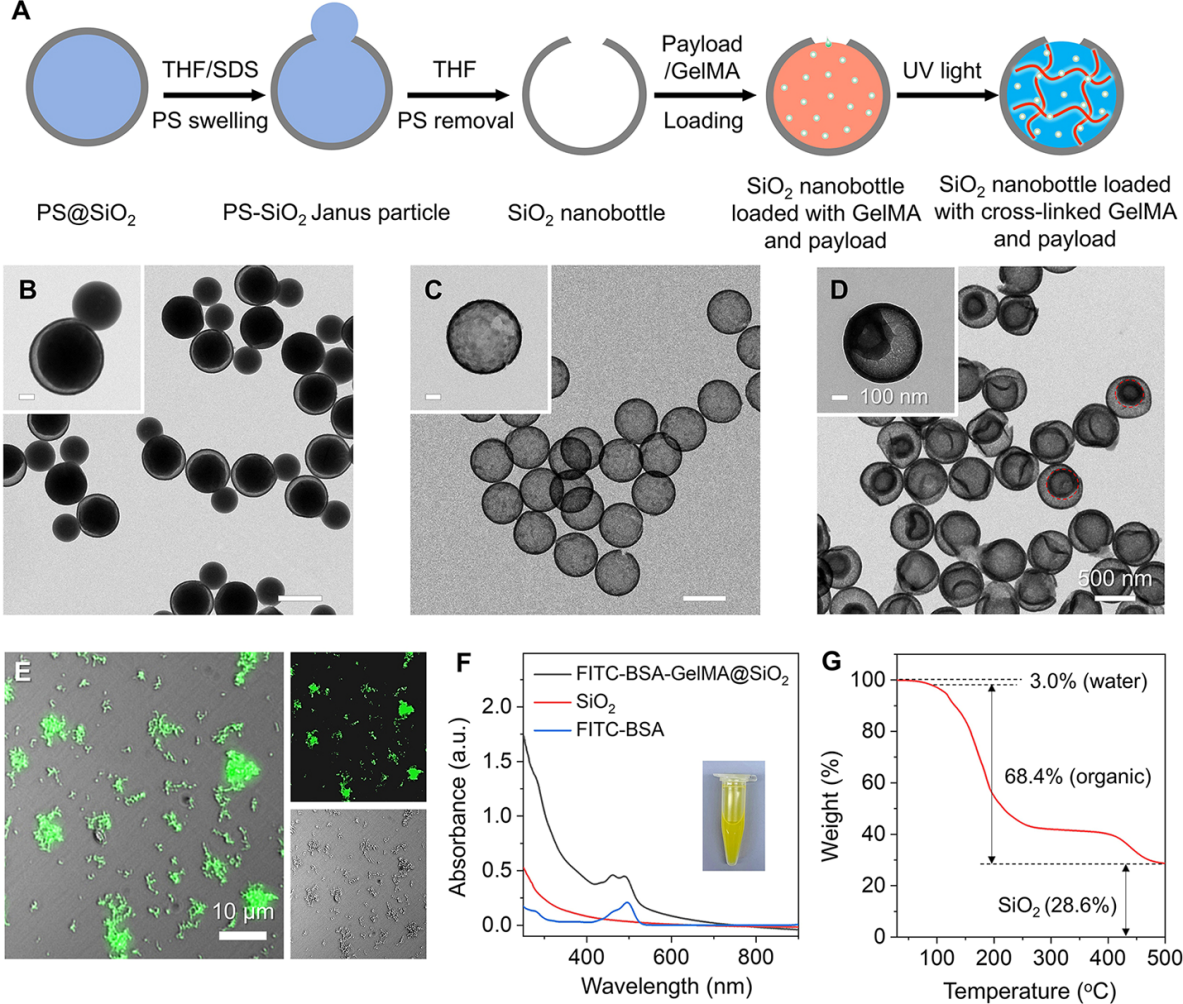
Synthesis, loading, and characterizations of SiO_2_ nanobottles. (A) Schematic showing the synthesis of SiO_2_ nanobottles loaded with cross-linked GelMA and a payload. (B–D) TEM images of PS-SiO_2_ Janus particles, empty SiO_2_ nanobottles, and SiO_2_ nanobottles loaded with GelMA and FITC-BSA (a model drug). The scale bars in the insets are 100 nm, while all panels share the same scale bar. (E) Merged fluorescence micrograph of SiO_2_ nanobottles after their cavity had been loaded with GelMA and FITC-BSA. The green fluorescence emitted from FITC-BSA upon excitation indicates their successful loading into the nanobottles. (F) Ultraviolet–visible (UV–vis) spectra recorded from an aqueous solution of FITC-BSA, as well as suspensions of empty nanobottles and the nanobottles filled with cross-linked FITC-BSA-GelMA. (G) Thermogravimetric analysis (TGA) analysis of the cross-linked nanobottles. The 68.4% weight loss up to 500 °C corresponds to the decomposition of the loaded GelMA and FITC-BSA.

To confirm the spatial distribution, we replaced FITC-BSA with a Ru-based compound and analyzed by scanning electron microscopy coupled with energy-dispersive X-ray spectroscopy (SEM-EDX) elemental mapping (Figure S2). The Ru signal was uniformly distributed throughout the nanobottle interior and colocalized with the carbon signal from the GelMA matrix, confirming internal encapsulation. UV–vis spectra showed a characteristic absorption peak at 450–495 nm, distinct from that of free FITC-BSA, likely arising from electronic interactions with the GelMA precursor (Figure 1F). TGA revealed an organic content (GelMA + payload) of 68.4 wt %, further confirming the successful incorporation of the polymer matrix within the nanobottles (Figure 1G). After UV cross-linking at 4 °C for 2 h, a cross-linked GelMA network was formed inside the nanobottles (denoted cross-linked FITC-BSA-GelMA@SiO_2_). Differential scanning calorimetry (DSC) analysis showed a ca. 5 °C red shift in the endothermic peak, indicating enhanced thermal stability of GelMA upon successful cross-linking (Figure S3). Fourier-transform infrared spectroscopy (FTIR) further confirmed >95% methacryloyl conversion, as evidenced by the decreased intensity of the characteristic C=C stretching band at 1635 cm^–1^ (Figure S4). TEM images in Figure S5 showed that the cross-linked GelMA remains stable under electron beam irradiation. The encapsulation efficiency (EE) of the payload was determined by comparing the amount of biomolecule encapsulated within the GelMA@SiO_2_ nanobottles to the total amount added (Table S1). However, the measured EE values were relatively low (3.2%–7.5%). Future optimization of loading and separation steps, as well as increasing the payload concentration or nanobottle quantity, could further enhance EE while preserving biomolecular integrity.

We evaluated the release profile of FITC-BSA from the cross-linked and non-cross-linked samples by incubating them in PBS at 37 °C and deriving the concentration of released FITC-BSA using UV–vis spectroscopy (Figure S6). The cross-linked group showed a relatively slow-release rate, with only ca. 14.0% of the FITC-BSA being released in the initial 3 days and ca. 51.2% being released over 42 days (Figure 2A). In contrast, the non-cross-linked group displayed a much faster release, with approximately 30.3% of the FITC-BSA being released within the first 3 days and over 80% over 42 days. To better understand the release kinetics, the cumulative release data were analyzed using the Korsmeyer–Peppas model (*F* = *kt* ^*n*^), where *F* is the fractional release at time *t*, *k* the kinetic constant, and *n* the release exponent indicative of the transport mechanism.^26,27^ As shown in Figure S7, non-cross-linked GelMA@SiO_2_ nanobottles displayed non-Fickian (anomalous) transport, suggesting contributions from both diffusion and polymer relaxation, while cross-linked nanobottles exhibited predominantly diffusion-controlled (Fickian) release. The *t*_1/2_ of FITC-BSA from the cross-linked group was 37 days, nearly five times longer than that of the non-cross-linked group. Such an extended half-life can be attributed to the restricted diffusion and thus slower release of the payload from a cross-linked network. Typically, non-cross-linked GelMA behaved like a viscous liquid at 37 °C,^28^ characterized by the loss of structural integrity and thus a faster release profile of the encapsulated payload from the opening. More importantly, unlike previously reported GelMA-based hydrogels and nanogels with a much shorter half-life of 1–3 days,^29–31^ the SiO_2_ solid wall could significantly prolong payload release by acting as a physical barrier to minimize the direct interaction between the payload and the external medium.

**Figure 2:**
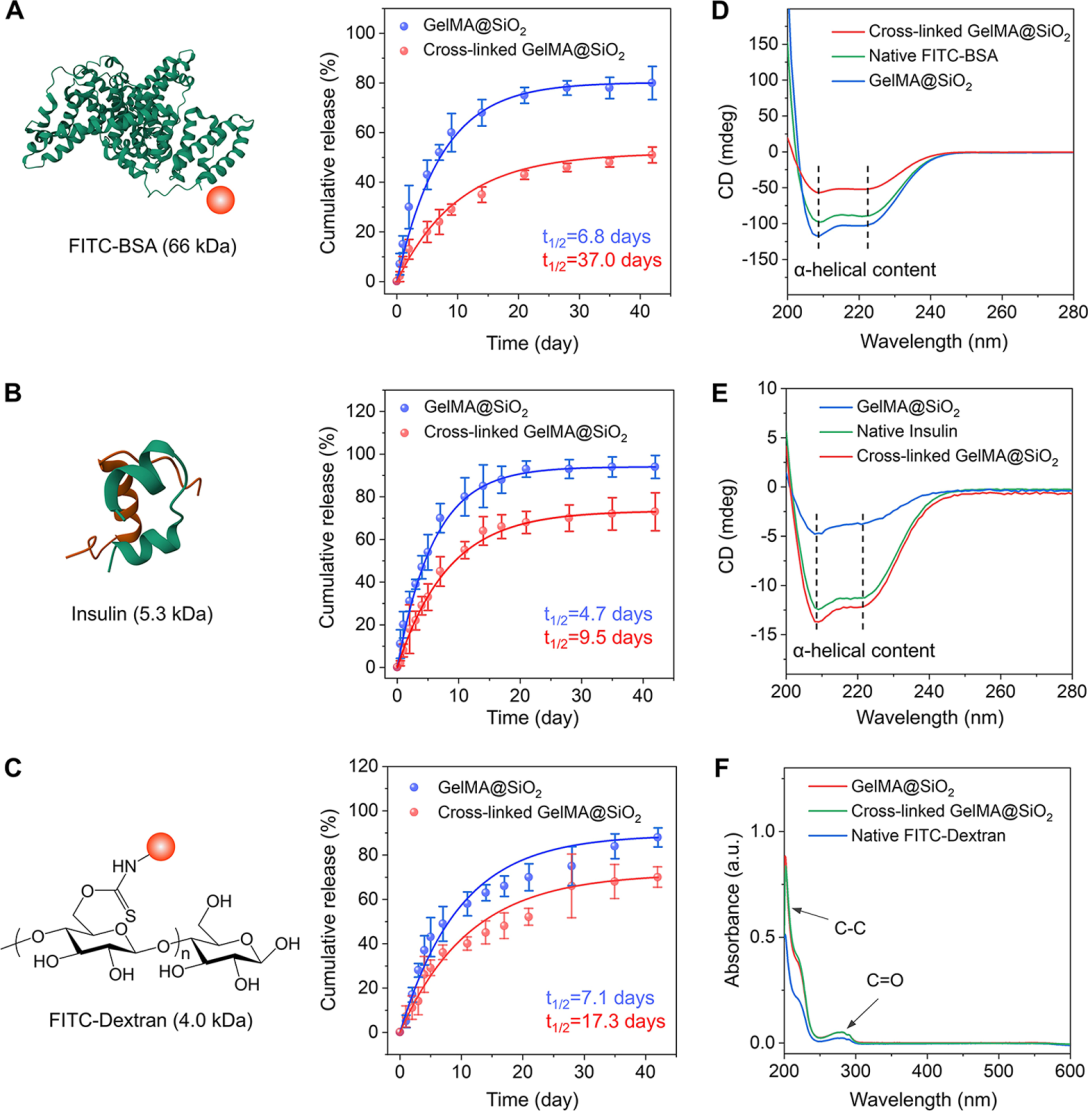
Photo-cross-linking of the GelMA matrix helps slow down the release of a payload from the nanobottles. Release kinetics in phosphate-buffered saline (PBS) (pH 7.4) measured for (A) FITC-BSA, (B) insulin, and (C) FITC-dextran loaded in SiO_2_ nanobottles, together with non-cross-linked and cross-linked GelMA. In particular, the red ball represents the fluorescent FITC moiety grafted to BSA or dextran. (D, E) CD spectra of FITC-BSA and insulin released from the non-cross-linked and cross-linked nanobottles, with comparison to their native forms. (F) UV-vis spectra of aqueous solution of FITC-dextran and those released from the non-cross-linked and cross-linked nanobottles.

Many reports have demonstrated that the release profile of a nanocarrier also depends on the characteristics of the payload, such as molecular weight, hydrodynamic radius, and hydrophobicity or hydrophilicity.^32–35^ To gain insights into these factors, we replaced FITC-BSA with insulin (Mw ≈ 5.3 kDa, a protein hormone) and FITC-dextran (Mw ≈ 4.0 kDa, a polysaccharide), and then measured the release profiles using UV–vis spectroscopy (Figures S8 and S9). For non-cross-linked samples, both payloads showed similar rapid release with a half-life of less than 1 week (Figures 2B and 2C), suggesting diffusion through the opening in the wall served as the rate-determining step. Upon cross-linking, insulin and FITC-dextran exhibited extended half-lives of 9.5 and 17.3 days, respectively. Although insulin and FITC-dextran share similar molecular weights, insulin, due to its more flexible backbone and smaller hydrodynamic radius, has a significantly larger diffusion coefficient through the cross-linked GelMA matrix. Zeta potential analysis indicates that electrostatic interactions also contribute to the distinct release behaviors of different payloads. Under physiological pH, insulin exhibited a more negative surface charge than both FITC-dextran and the SiO_2_ nanobottle matrix, resulting in stronger electrostatic repulsion and faster diffusion through the cross-linked GelMA network (Table S2). In contrast, the nearly neutral FITC-dextran experienced weaker electrostatic effects and was more confined within the hydrogel mesh. These combined steric and electrostatic effects explain the faster release of insulin than FITC-dextran, despite their similar molecular weights, highlighting the interplay between cross-linking density, molecular structure, and surface charge in controlling the release kinetics.

The structural integrity of the released proteins was examined by circular dichroism (CD) spectroscopy (Figures 2D and 2E). The CD spectra of FITC-BSA and insulin released from both non-cross-linked and cross-linked samples closely resembled those of their native forms, with characteristic double minimum at 209 and 222 nm corresponding to α-helical structures. UV-vis spectra of released FITC-BSA showed the same characteristic peaks at 280 and 495 nm, corresponding to the C=O group from BSA and the fluorescein chromophore from FITC (Figure S10). Similarly, the UV–vis spectra of the released FITC-dextran displayed strong absorption below 200 nm and a peak at 280 nm. They could be attributed to C–C and C=O bonds, respectively, and were consistent with those of native FITC-dextran in a solution (Figure 2F). All these results indicate that the structure and functional domain of the payloads remain intact throughout the loading and release cycle. Taken together, we can conclude that the payloads can be controlled to release over an extended period while retaining their functional structures throughout the process. The sustained release, coupled with the preservation of structures, makes the current system promising for applications that demand long-term, controlled release of bioactive molecules.

Although the released payloads retained their structural stability, their functionality and practical efficiency remained unknown. To this end, we used lysozyme (Mw ≈ 14 kDa) as a model drug to assess its bioactivity upon release from the nanobottles. The release profiles of lysozyme from both non-cross-linked and cross-linked samples follow a trend similar to that observed for FITC-BSA (see Figure 3A, as well as Figure S11). After 7 days, the cumulative release from the cross-linked group was significantly less than that from the non-cross-linked group (ca. 30% vs ca. 50%). We then assessed the antimicrobial effectiveness of the released lysozyme using*Micrococcus lysodeikticus*cells, a well-established model for testing lysozyme activity. Lysozyme hydrolyzes the peptidoglycan layer of bacterial cell walls, leading to cell lysis, which was monitored by recording absorbance at 450 nm in Dulbecco’s PBS (DPBS) using a microplate reader. The slopes of the absorbance–time plots were compared to that of native lysozyme to determine relative bioactivity.

**Figure 3:**
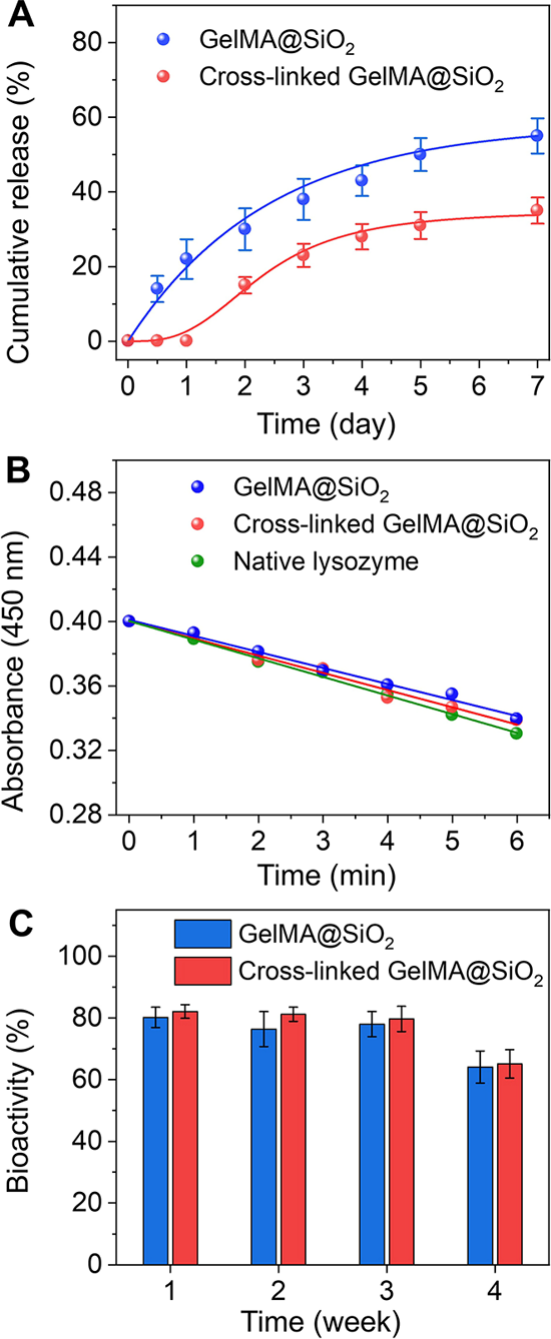
Characterizations of the bioactivity of lysozyme released from the nanobottles. (A) Release profiles of lysozyme in PBS (pH 7.4) from non-cross-linked and cross-linked nanobottles, respectively. We measured the concentration of the released lysozyme by recording the absorbance at 562 nm using the Micro BCA protein assay. (B) Linear fit (where *y* is absorbance and *x* is time) for the native lysozyme (*y* = −0.011*x* + 0.400, *R* ^2^ = 0.99), lysozyme released from the non-cross-linked (*y* = −0.0099*x* + 0.401, *R* ^2^ = 0.99), and the cross-linked (*y* = −0.0107*x* + 0.400, *R* ^2^ = 0.98) nanobottles. (C) Comparison of the bioactivity for the lysozyme released from the non-cross-linked and cross-linked nanobottles over a period of 4 weeks.

As shown in Figure 3B, the lysozyme released from the non-cross-linked and cross-linked samples retained about 80% ± 3% and 82% ± 2% of its bioactivity, respectively. These data indicated that the lysozyme released from both groups exhibited higher bioactivity, compared to other types of carriers reported in the literature, such as colloidal emulsions or poly­(lactic-co-glycolic acid) microspheres,^36,37^ which showed bioactivity levels between 30% and 70%. The retention of bioactivity highlights the effectiveness of the current system in preserving the functional integrity of the encapsulated enzymes. Over the subsequent three weeks, a gradual decline in lysozyme bioactivity was observed. Specifically, the bioactivity of lysozyme from the non-cross-linked group decreased to 76% ± 5%, 78% ± 4%, and 64% ± 5% at weeks 2, 3, and 4, respectively (Figure 3C). Similarly, for the cross-linked group, the bioactivity dropped to 81% ± 2%, 79% ± 4%, and 65% ± 4% over the same period. Despite some decrease, the lysozyme released from both groups maintained a higher level of bioactivity over time compared to traditional delivery systems, underscoring the potential of nanobottles for applications involving long-term storage and delivery of enzymes.

To assess the cytocompatibility of the nanocarrier system, a cell counting kit-8 (CCK-8) assay was performed on A549 cells after 3 days of incubation with blank nanobottles, as well as nanobottles loaded with non-cross-linked or cross-linked GelMA. As shown in Figure S12, all groups exhibited cell viability comparable to the untreated control, confirming negligible cytotoxicity. These results demonstrate the excellent biocompatibility of both the nanobottle and GelMA formulation.

Because of cross-linking and retarded release, we argued that the nanobottles with cross-linked GelMA would exhibit enhanced intracellular stability. To validate this argument, we incubated both non-cross-linked and cross-linked samples containing FITC-BSA with A549 cells for 24 h and imaged by confocal microscopy (see Figures 4A and 4B). The non-cross-linked sample displayed a diffusive fluorescence pattern, indicating the rapid, premature release of FITC-BSA (Figure 4A). In contrast, as shown in Figure 4B, the cross-linked sample exhibited a distinct dotlike fluorescence pattern surrounding the nucleus, suggesting superior intracellular stability and a delayed release of FITC-BSA.^38^ The enhancement in stability could be attributed to the cross-linked network, which significantly retarded the diffusion of the payload. Fluorescence line-scan profiles further supported this observation: the cross-linked group exhibited multiple sharp green peaks (corresponding to FITC-BSA), while the non-cross-linked group showed a broad, uniform signal (Figures 4C and 4D). These results confirm that cross-linking the GelMA matrix could delay intracellular release by reducing molecular diffusion. Figure 4E illustrates a schematic comparison of the stability of the cross-linked and non-cross-linked nanobottles during the internalization process. Without cross-linking, the SiO_2_ shell only offered minimal retention, allowing the payload to diffuse out even before the nanobottle was fully internalized by the cell. In contrast, the cross-linked GelMA matrix could significantly stabilize the payload, enabling sustained and localized intracellular release. Taken together, it can be concluded that cross-linking of GelMA enhances the efficiency of intracellular delivery by slowing down molecular diffusion and reducing the risk of premature release, thereby enabling more effective delivery.

**Figure 4:**
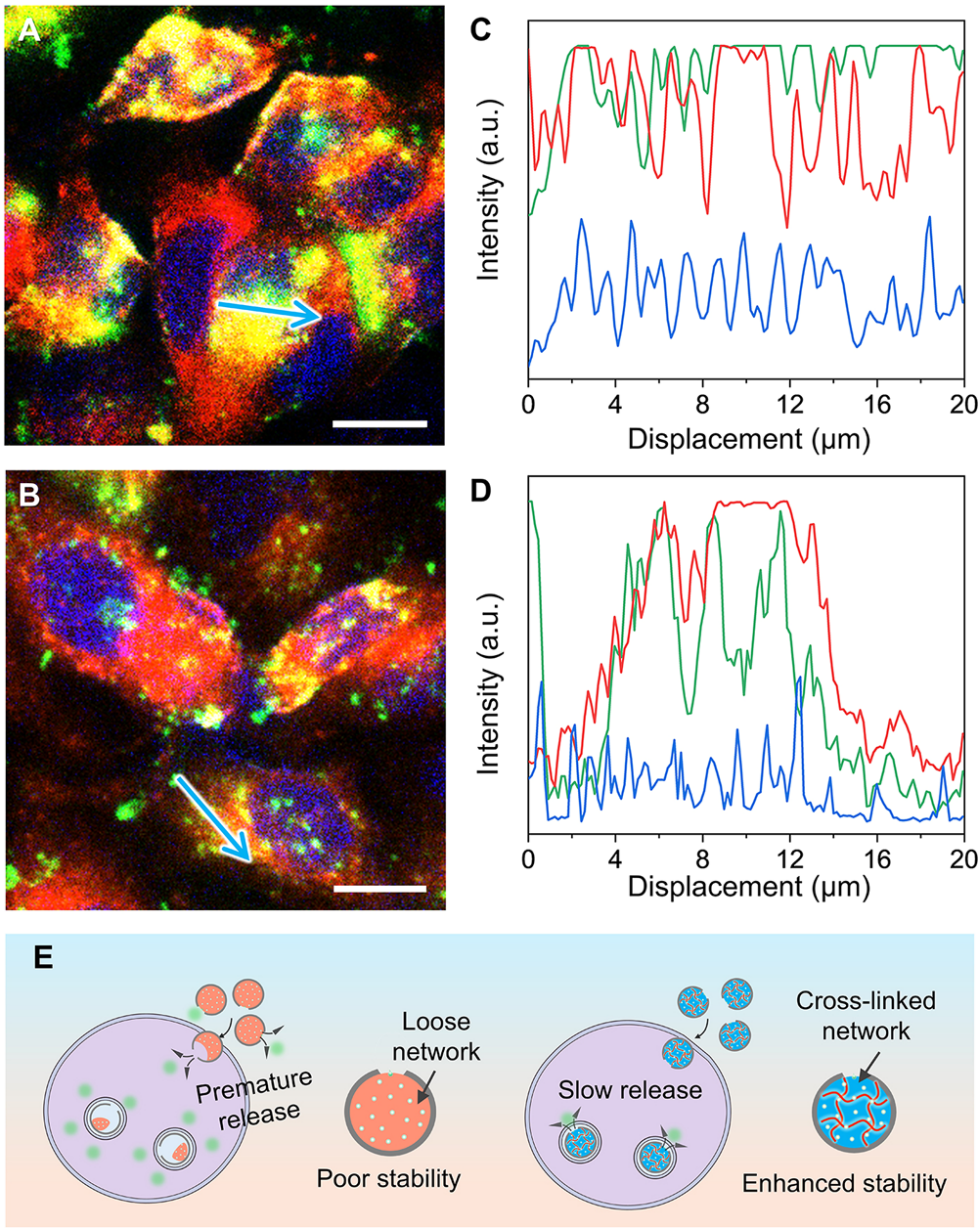
Comparative analysis of cellular behaviors of FITC-BSA-GelMA@SiO_2_ nanobottles (with or without cross-linking) using A549 cells. Representative fluorescence images of A549 cells after culture with nanobottles containing (A) non-cross-linked and (B) cross-linked GelMA loaded with FITC-BSA (ca. 0.1 mg mL^–1^), respectively, for 24 h. The cell nucleus was stained with Hoechst 33342 (blue) and lysosomes were stained with LysoTracker (red), whereas FITC-BSA gave a green color. Scale bars = 10 μm. (C, D) Fluorescence intensity line profiles analyses across representative cells in the non-cross-linked and cross-linked groups, respectively, showing the spatial distribution of FITC-BSA (green), lysosomes (red), and nucleus (blue). The intensities were measured along the lines marked with white arrows on the fluorescence images. (E) Schematic illustrating the effect of cross-linking on the release behavior during the cell culture study (left side) without and (right side) with cross-linking of the GelMA matrix.

To investigate the impact of the sustained release of biological factors from nanobottles on neurite extension, we switched to nerve growth factor (NGF) as the payload,^39^ and examined the influence of the released NGF on neurite outgrowth from PC12 cells in vitro. The fluorescence micrographs in Figures 5A–C, confirm neurite projections from PC12 cells after 7 days of incubation. The cells treated with plain, cross-linked nanobottles exhibited a rounded morphology with minimal neurite initiation, indicating the absence of NGF stimulation. In contrast, cells exposed to NGF-loaded, non-cross-linked GelMA@SiO_2_ nanobottles displayed moderate morphological changes and neurite extension, due to the rapid, burst release of NGF from the nanobottles. Notably, the cells treated with NGF-loaded, cross-linked GelMA@SiO_2_ nanobottles exhibited significant neurite outgrowth with longer and more-defined projections, suggesting a sustained and bioactive release of NGF during culture. Quantitative analysis of neurite length (Figure 5D) confirmed these observations. The average neurite length in the cross-linked group (73.1 ± 5.7 μm) was significantly greater than that in the non-cross-linked group (25.1 ± 1.6 μm) and the NGF-absent group (5.1 ± 2.3 μm), highlighting the importance of controlled release of NGF in promoting neural differentiation and neurite extension. The enhanced performance of the cross-linked group is primarily attributed to the sustained and controlled release of NGF from the cross-linked GelMA matrix encapsulated in the nanobottles. The cross-linked network offers a more stable structure, enabling prolonged and gradual release of NGF, helping maintain effective local concentrations that provide continuous stimulation conducive to neurite elongation.^40^ To further verify the release profile, NGF solutions were collected from both non-cross-linked and cross-linked samples (ca. 4.0 mg) after 3 days of incubation in the culture medium at 37 °C. The NGF concentration released from the non-cross-linked sample reached 0.473 μg mL^–1^, which was 2.5 times greater than that from the cross-linked group (0.135 μg mL^–1^), confirming the slower release kinetics of the cross-linked system (Figure S13). Altogether, these findings demonstrate that neurite extension is significantly enhanced by the slow and sustained release of NGF from SiO_2_ nanobottles containing a cross-linked hydrophilic network.

**Figure 5:**
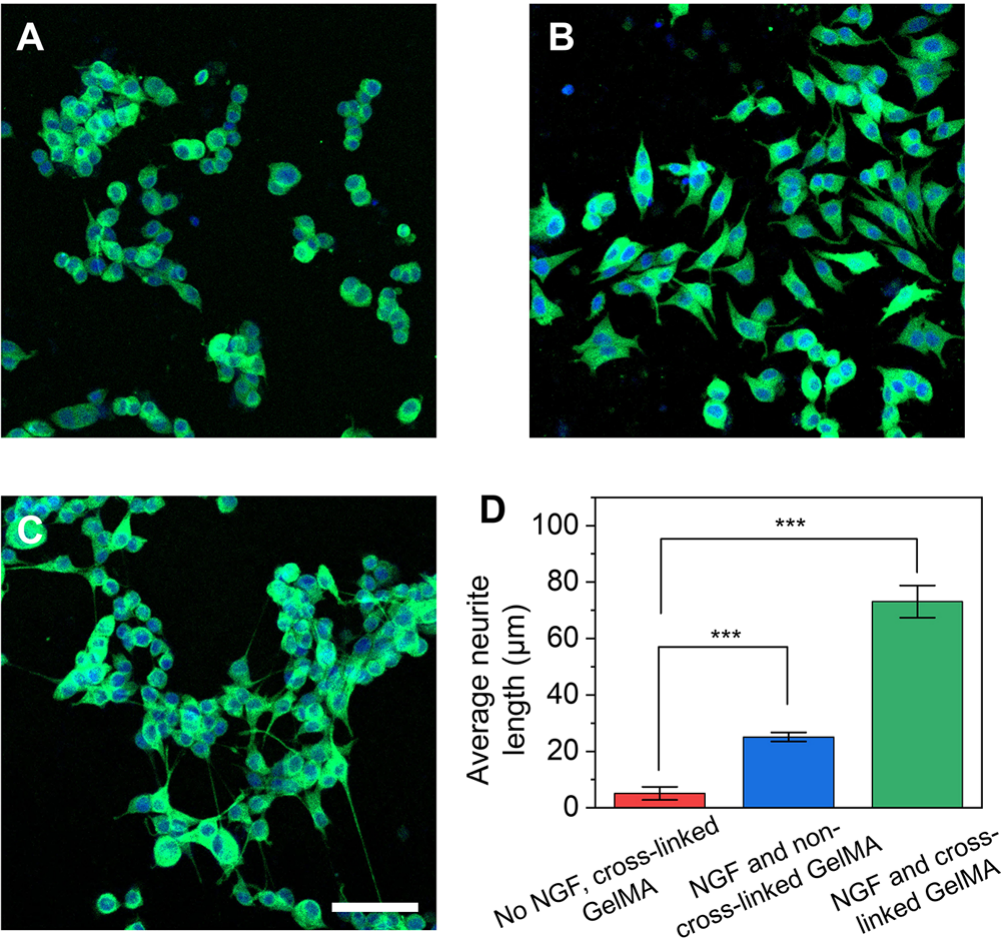
Neurite outgrowth from PC12 cells in response to NGF released from the nanobottles. Fluorescence micrographs of the neurite extension from PC12 cells cultured on a laminin-coated 24-well plate for 7 days. The cells were treated with nanobottles loaded with (A) cross-linked GelMA only (no NGF), (B) NGF and non-cross-linked GelMA, and (C) NGF and cross-linked GelMA. The neurites were stained with Tuj1 marker (green) whereas the nuclei were labeled with DAPI (blue). Scale bars = 50 μm. (D) Average neurite length of extended neurites from the PC12 cells when cultured with (i) no NGF-loaded, cross-linked nanobottles, (ii) NGF-loaded, non-cross-linked nanobottles, and (iii) NGF-loaded, cross-linked nanobottles. (***) *p* < 0.001.

In summary, we have developed a hybrid system that integrates SiO_2_ nanobottles with photo-cross-linked GelMA to achieve sustained release of hydrophilic biomacromolecules such as proteins, enzymes, and polysaccharides. We can regulate the release kinetics and extend the half-life of the encapsulated payloads from several days to weeks. In vitro analysis demonstrated that the hybrid system showed enhanced intracellular stability, effectively preventing burst release while allowing for controlled release inside the cell. In the context of nerve repair, the sustained release of nerve growth factor facilitated neurite outgrowth in PC12 cells. While this study focuses on proteins, enzymes, and polysaccharides, the protective hydrogel matrix and the nanobottle confinement should also work with nucleic acids such as mRNA and plasmid DNA,^41,42^ which are increasingly important therapeutic agents. The negatively charged backbone of nucleic acids could favorably interact with the amine-rich GelMA network or the silanol groups on the SiO_2_ surface, enhancing their retention and thereby protection against degradation.^43^ Given its versatility, biocompatibility, and controlled release capability, this hybrid platform holds promise for advanced drug delivery applications, offering a robust solution for burst-free, long-term therapeutic administration.

## Supplementary Material



## Notes

Experimental section; SEM-EDX mapping, DSC curves, and TEM images of GelMA@SiO_2_ nanobottles; FTIR spectra of non- and cross-linked GelMA; calibration curves and cumulative release profiles of FITC-BSA, insulin, FITC-dextran, and lysozyme; UV-vis spectra of the FITC-BSA released from the non-cross-linked and cross-linked GelMA@SiO_2_ nanobottles; concentration of NGF released from the non-cross-linked and cross-linked nanobottles; cell viability of A549 cells; encapsulation efficiency of different payloads in the GelMA@SiO_2_ nanobottles; zeta potential of SiO_2_ nanobottles and three representative payloads (PDF)
