# Supplementary material for: Silica Nanobottles Filled with Photo-cross-linked GelMA for the Sustained Release of Hydrophilic Biomacromolecules
Source: Nano Lett. 2025 Nov 24;25(49):17050–7. doi: 10.1021/acs.nanolett.5c04288 (PMC12874627; doi:10.1021/acs.nanolett.5c04288)
Supplement: Supplementary file 1 [file nl5c04288_si_001.pdf]

Supporting Information

Silica Nanobottles Filled with Photo-cross-linked GelMA for the Sustained Release of Hydrophilic Biomacromolecules

Dong Zhang,[†,¶] Yuxuan Meng,[§,¶] and Younan Xia[†,§,*]

[†]The Wallace H. Coulter Department of Biomedical Engineering, Georgia Institute of Technology and Emory University, Atlanta, Georgia 30332, United States

[§]School of Chemistry and Biochemistry, Georgia Institute of Technology, Atlanta, Georgia 30332, United States

[¶]These authors contributed equally to this work.

*Corresponding author: younan.xia@bme.gatech.edu

EXPERIMENTAL SECTION

Chemicals and materials. Tetraethylorthosilicate (TEOS, 98%), poly(allylamine hydrochloride) (PAH, Mw≈50,000), poly(vinyl pyrrolidone) (PVP, Mw≈360,000), sodium dodecyl sulfate (SDS, 99%), sodium chloride (NaCl), Ruthenium(III) chloride hydrate, aqueous ammonia (28-30%), tetrahydrofuran (THF, 99.9%), gelatin methacryloyl (GelMA, 40% of substitution), fluorescein isothiocyanate-conjugated bovine serum albumin (FITC-BSA, Mw≈66 kDa), FITC-dextran (Mw≈4.0 kDa), 2-hydroxy-4'-(2-hydroxyethoxy)-2-methylpropiophenone (Irgacure 2959, 98%), lysozyme from chicken egg white (>98%), and insulin from bovine pancreas (Mw≈5.3 kDa) were purchased from Sigma-Aldrich and used as received. Polystyrene (PS) beads with an average diameter of 500 nm were obtained from Polysciences. Phosphate-buffered saline (PBS, pH=7.4) buffer, 4', 6-diamidino-2-phenylindole (DAPI), Hoechst 33342, and human beta-nerve growth factor recombinant protein (NGF, Mw≈27 kDa, >98%) were ordered from Thermo Fisher Scientific. Cell Counting Kit-8 was obtained from Dojindo. Deionized water with a resistivity of 18.2 MΩ·cm at room temperature was used in all experiments.

Preparation of the PS@SiO₂ core-shell particles. The particles were synthesized according to a published protocol with some modifications.[1] Typically, 0.5 mL of a 2.5% (*w*/*v*) aqueous suspension of PS beads was washed three times with water, followed by re-dispersion in 5 mL of an aqueous solution containing NaCl (1.5 mg mL^{-1}) and PAH (1.5 mg mL^{-1}). The mixture was incubated on a shaker (300 rpm) at 25 °C for 1 h. Subsequently, the pretreated particles were washed twice with water before being dispersed in 2.5 mL of PVP (7 mg mL^{-1}) solution in ethanol. After shaking for another 1 h, the particles were washed with ethanol and then redispersed in 5 mL of ethanol. To form a uniform shell made of SiO_2, 400 μL of aqueous ammonia and 15 μL of TEOS were sequentially added, and the coating process was allowed to proceed at room temperature overnight. The resulting solid products were collected *via* centrifugation (8,000 rpm, 5 min), washed three times with ethanol, and eventually re-dispersed in 1 mL of water for further use.

Swelling of the PS@SiO₂ particles with a THF/SDS mixture. We mixed 0.4 mL of THF and 0.6 mL of the 1% (*w*/*v*) aqueous SDS solution to obtain a mixture. SDS was introduced to help stabilize the colloidal suspension. We then mixed 1 mL of the THF/SDS mixture with 50 μL of the aqueous suspension of PS@SiO_2 particles followed by incubation on a shaker (300 rpm) at room temperature. After 10 h, 5 mL of ethanol was introduced to quench the swelling process. The solid

products (denoted PS-SiO_2 Janus particles) were collected *via* centrifugation (8,000 rpm, 5 min) and sequentially washed with ethanol and water.

Preparation of the SiO_2 nanobottles. We dispersed 50 μL of the aqueous suspension of the PS-SiO_2 Janus particles in 1.0 mL of THF, followed by incubation on a shaker (300 rpm) at room temperature overnight. The resulting solid products were collected *via* centrifugation (10,000 rpm, 5 min), washed with THF three times, and sequentially washed with ethanol and water.

Loading GelMA and a hydrophilic molecular payload into SiO_2 nanobottles. Typically, we mixed 40 μL of a suspension of the SiO_2 nanobottles with 1.0 mL of 15 mg mL^{-1} GelMA in PBS solution containing 5 mg of a payload (*e.g.*, FITC-BSA) and 0.2 mg of Irgacure 2959 (photo-initiator), followed by shaking (300 rpm) at 37 °C for 6 h to fill the nanobottles with the GelMA and payload. The solid products were collected through centrifugation (10,000 rpm, 5 min) and washed with PBS solution (37 °C) twice. In similar attempts, we substituted FTIC-BSA with FITC-dextran, insulin, lysozyme, or NGF to obtain FITC-dextran-GelMA@SiO_2, insulin-GelMA@SiO_2, lysozyme-GelMA@SiO_2, or NGF-GelMA@SiO_2 nanobottles. Subsequently, the suspension of loaded nanobottles was exposed to UV irradiation (4 W, 365 nm) for 2 h to cross-link the GelMA at 4 °C. A distance of 20 mm was consistently maintained between the UV lamp and the suspension throughout the experiment. The drug encapsulation efficiency (EE) was determined utilizing the equation below:

$$EE = \frac{W_0}{W_{drug}} * 100\%$$

where W_0 and W_{drug} represent the weight of the drug in nanobottles and the weight of the drug added, respectively (Table S1).

Measurement of release profiles. 0.5 mL of the FITC-BSA-loaded nanobottles (non- or cross-linked) in PBS solution was transferred to a 1.5-mL centrifuge tube. The sample was placed on a shaker (250 rpm) at 37 °C. At designated time points (0.5, 1, 2, 5, 7, 9, 14, 21, 28, 35, and 42 days), the sample was cooled in an ice bath for 5 min and then centrifuged at 8000 rpm for 6 min. A 0.2-mL aliquot of the supernatant was collected for UV-vis measurement. After measurement, the collected supernatant was returned to the centrifuge tube to maintain the release environment. To determine the total loading amount of FITC-BSA, 0.5 mL fresh suspension of FITC-BSA-loaded GelMA@SiO_2 nanobottles (non-cross-linked) was heated at 60 °C for 15 min, centrifuged at 8000 rpm for 6 min, and the supernatant was analyzed *via* UV spectroscopy. Each concentration was determined using the calibration curve in Figure S5, and the release profile of FITC-BSA was

established. For the release profiles of other loaded biomacromolecules, including insulin and FITC-dextran, the same procedure was followed with slight adjustments to the designated time points. Their corresponding calibration curves are provided in Figures S7-8.

Characterizations. The samples were characterized by using a transmission electron microscope (TEM, Hitachi 7700) operated at 120 kV. The spatial distribution of the payload within the nanobottles was examined using scanning electron microscopy coupled with energy-dispersive X-ray spectroscopy (SEM-EDX, Hitachi SU8230). To determine the content of loading, we characterized the FITC-BSA-GelMA@SiO_2 nanobottles using thermal gravimetric analysis (TGA) with a Mettler Toledo analyzer. Briefly, the dried particles (*ca.* 2 mg) were added to a platinum pan, which was then placed on the sample holder. The system was equilibrated at 25 °C for 5 min, followed by TGA measurement with a temperature ramp of 20 °C min^{-1} up to 500 °C under an argon atmosphere. Differential scanning calorimetry (DSC) was recorded by a Discovery DSC250 (TA Instruments, USA) under an N_2 atmosphere. The as-prepared GelMA@SiO_2 nanobottles (*ca.* 2.0 mg) before and after photo-cross-linking were placed in a hermetic aluminum pan, equilibrated at -80 °C, and then heated at a ramping rate of 10 °C min^{-1} up to 70 °C. The UV-vis spectra were recorded on a Cary 60 spectrometer (Agilent Technologies). Fourier transform infrared (FTIR) spectroscopy (IRAffinity-1, Shimadzu, Kyoto, Japan) was performed to monitor the conversion of methacryloyl C=C groups and confirm GelMA cross-linking within the nanobottles. For fluorescence microscopy, the sample was prepared by drying a drop of the particle suspension on a glass slide and characterized using a laser confocal scanning microscope (Zeiss LSM 900, Carl Zeiss, Germany). Circular dichroism (CD) spectra were recorded using a Chirascan CD spectrophotometer (Applied Photophysics) at a scanning speed of 1 nm s^{-1} (200–280 nm). The testing temperature was set to 20 °C.

Calculating the concentration of the released lysozyme. Native lysozyme was dissolved in Dulbecco's PBS (DPBS) to obtain solutions of 1, 2.5, 7.5, 10, 15, and 20 μg mL^{-1} in concentration. Micro BCA Protein Assay (0.1 mL) was added to 0.1 mL of the lysozyme sample with a known (native) or an unknown (released from the nanobottles) concentration. The culture tubes were incubated at 60 °C for 1 h and then examined with a spectrometer. From the known samples, we could obtain a calibration curve by plotting the absorbance at 562 nm against the concentration of lysozyme. The unknown concentrations of the released lysozyme were then determined from the calibration curve.

Bioactivity test for the released lysozyme. The bioactivity of lysozyme was determined from the rate of lysis for *Micrococcus lysodeikticus* cells. 0.1 mL of DPBS buffer containing 0.05 mg *Micrococcus Lysodeikticus* was added into a 96-well plate, followed by 0.1 mL of native or released lysozymes (at the same concentration). The plate was immediately placed into a microplate reader, and the absorbance at 450 nm was recorded every minute. We then plotted the absorbance at 450 nm against the reaction time for both native and released lysozymes. The ratio of the slopes for the two linear fits indicates the percentage of enzyme bioactivity after undergoing the loading and releasing processes.

Cytotoxicity Assay. A549 cells were cultured in Roswell Park Memorial Institute 1640 medium (RPMI 1640), supplemented with 10% fetal bovine serum (FBS) and 1% antibiotics (penicillin and streptomycin), at 37 °C in a humidified atmosphere with 5% CO_2. Cells were seeded in a 96-well plate at a density of 1.0×10^4 cells per well and cultured for 24 h. Subsequently, 10 μL of the suspension of blank, non-cross-linked, or cross-linked nanobottles (1.0 mg mL^{-1}) was added to each well, followed by incubation at 37 °C for 72 h. After incubation, the culture medium was replaced with serum-free RPMI 1640 containing 10% (v/v) Cell Counting Kit-8 solution, and the cells were incubated for an additional 1 h at 37 °C. The absorbance at 450 nm was then measured using a multi-mode plate reader (BioTek Synergy H1, Agilent Technologies, USA) to assess cell viability.

Intracellular stability of the cross-linked FITC-BSA-GelMA@SiO₂ nanobottles. A549 cells were cultured in RPMI 1640, supplemented with 10% FBS and 1% antibiotics (penicillin and streptomycin), at 37 °C in a humidified atmosphere with 5% CO_2. After reaching the optimal density, the A549 cells were seeded in a 24-well plate at a density of 4.0×10^4 cells per well and cultured for 24 h. 50 μL of the suspension of non-cross-linked and cross-linked nanobottles (1.0 mg mL^{-1}) were then added to each well, followed by incubation at 37 °C for 24 h. After washing with PBS once, the cells were stained with Lysotracker Red (75 nM) in PRMI 1640 at 37 °C for 15 min, followed by Hoechst 33342 (10 μg mL^{-1}) in RPMI 1640 at 37 °C for 10 min. Subsequently, the stained cells were washed with PBS three times. The fluorescence micrographs were captured using a confocal laser scanning microscope (Zeiss LSM 900) and further analyzed with Image-J software.

Culture of PC12 cells. PC12 cells were seeded at a density of 50–200 cells mm^{-2} in a 24-well plate using DMEM supplemented with 10% horse serum and 5% newborn calf serum. After culture

for 24 h, the medium was replaced with a differentiation medium consisting of DMEM supplemented with 1% horse serum and 1.0 mg mL^{-1} NGF-loaded GelMA@SiO_2 nanobottles (either cross-linked or non-cross-linked). Cells treated with DMEM containing 1% horse serum and 1.0 mg mL^{-1} GelMA@SiO_2 nanobottles without NGF served as the control group. After 7 days of incubation, neurites were immune-stained with anti-βIII tubulin antibody, and cell nuclei were stained with DAPI. The samples were imaged using a laser scanning confocal microscope. Fluorescence micrographs were analyzed with ImageJ software to quantify the average neurite lengths. Triplicate samples in each group were used for the study.

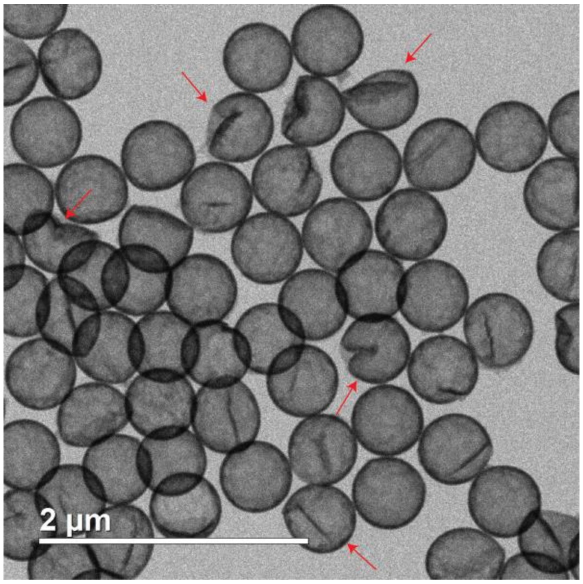

Figure S1. TEM image of SiO_2 nanobottles showing overall morphology and structural integrity. Arrows indicate defective or partially collapsed shells.

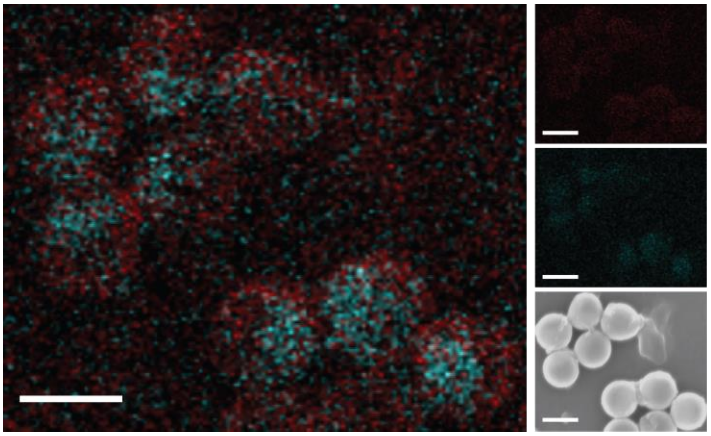

Figure S2. SEM-EDX elemental mapping of Ru (cyan) and C (red) signals in GelMA nanobottles. The uniform Ru distribution across the interior confirms homogeneous payload (*i.e.*, Ruthenium(III) chloride hydrate) loading within the GelMA matrix. Scale bars: 500 nm.

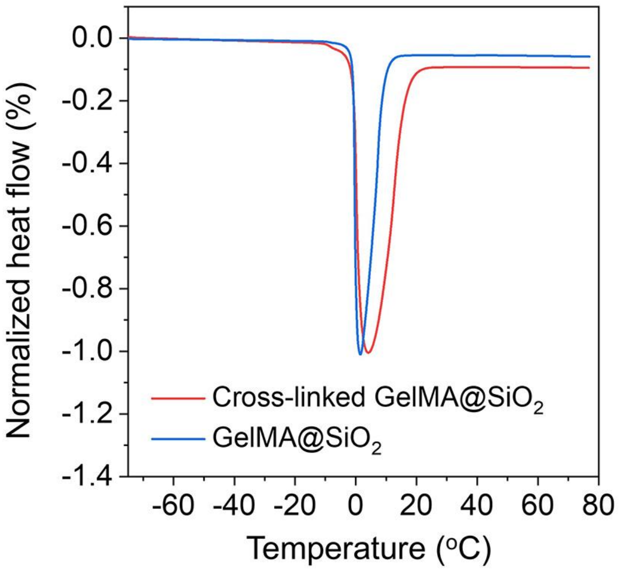

Figure S3. DSC curves of the FITC-BSA-GelMA@SiO_2 nanobottles with and without photo-cross-linking, respectively. The nanobottles (*ca*. 2.0 mg) were placed in a hermetic aluminum pan, equilibrated at -75 °C, and then heated at a ramping rate of 10 °C min^{-1} up to 70 °C.

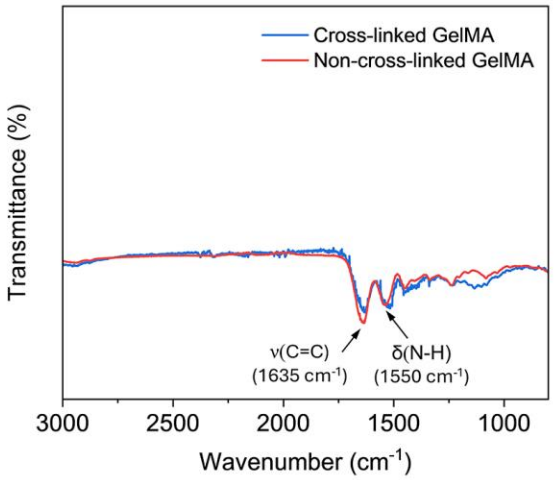

Figure S4. FTIR spectra of lyophilized cross-linked and non-cross-linked GelMA.

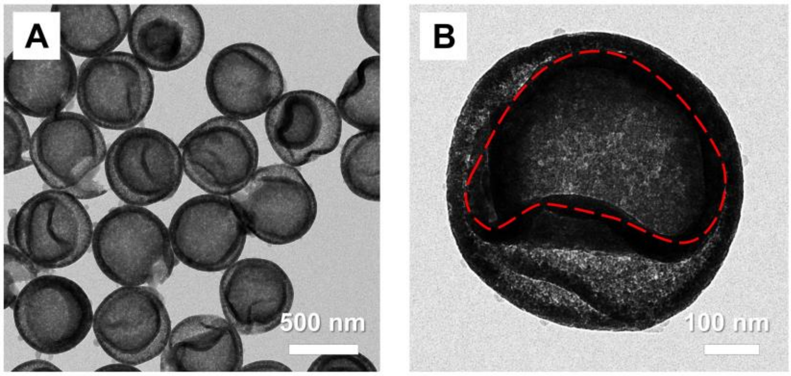

Figure S5. TEM images of the cross-linked FITC-BSA-GelMA@SiO_2 nanobottles. The red dotted circle in panel B marks the cross-linked GelMA. This image also demonstrates the good stability of the cross-linked GelMA within the nanobottle under the irradiation of electron beam.

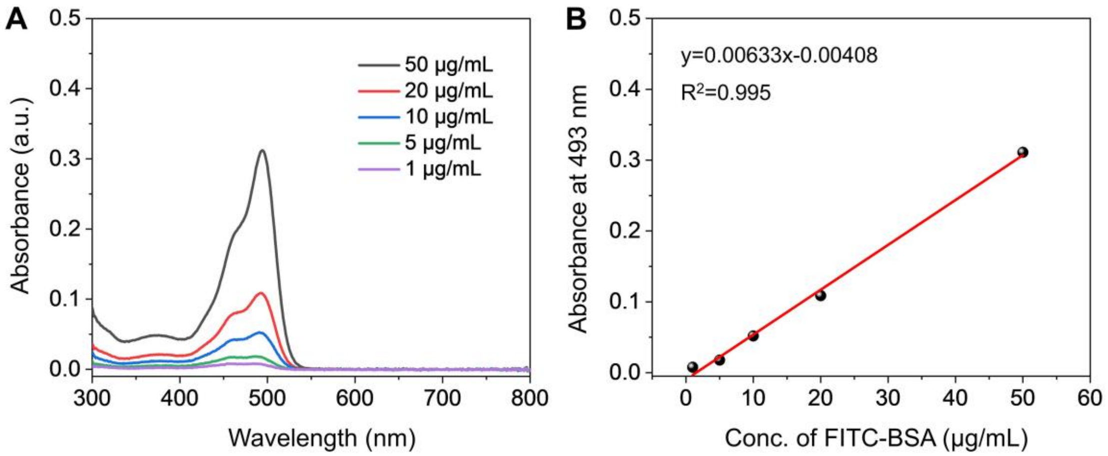

Figure S6. (A) UV-vis spectra of aqueous solutions of FITC-BSA at concentrations of 1–50 μg mL^{-1} and (B) the corresponding calibration curve used for determining the concentrations of FITC-BSA released from the nanobottles.

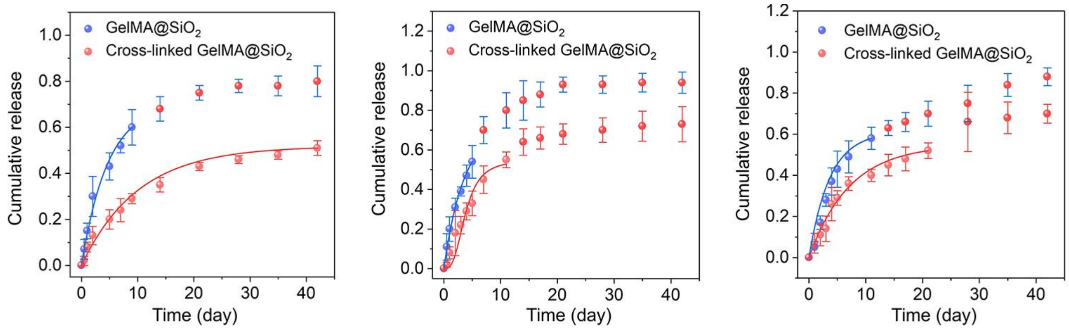

Payload (s)	Nanobottles	k value	n value	Mechanism (s)
FITC-BSA	GelMA@SiO$_2$	0.149 ± 0.026	0.643 ± 0.091	Non-Fickian (anomalous) transport
	Cross-linked GelMA@SiO$_2$	0.096 ± 0.010	0.466 ± 0.032	Fickian diffusion
Insulin	GelMA@SiO$_2$	0.195 ± 0.033	0.635 ± 0.145	Non-Fickian (anomalous) transport
	Cross-linked GelMA@SiO$_2$	0.073 ± 0.012	0.869 ± 0.077	Case-II transport; Non-Fickian (anomalous) transport
FITC-dextran	GelMA@SiO$_2$	0.107 ± 0.016	0.740 ± 0.078	Non-Fickian (anomalous) transport
	Cross-linked GelMA@SiO$_2$	0.120 ± 0.019	0.497 ± 0.063	Fickian diffusion

Figure S7. Cumulative release profiles of FITC-BSA, insulin, and FITC-dextran from GelMA@SiO$_2$ (blue) and cross-linked GelMA@SiO$_2$ (red) nanobottles over 42 days. Solid lines represent nonlinear fitting to the Korsmeyer-Peppas model (Release range: 0-60%). The fitting parameters (k and n values) and the corresponding release mechanisms are summarized in the table. Non-Fickian (anomalous) transport indicates a combined contribution of diffusion and polymer relaxation, while n values close to theoretical Fickian exponents suggest diffusion-controlled release, and n values approaching “1” indicate Case-II transport.

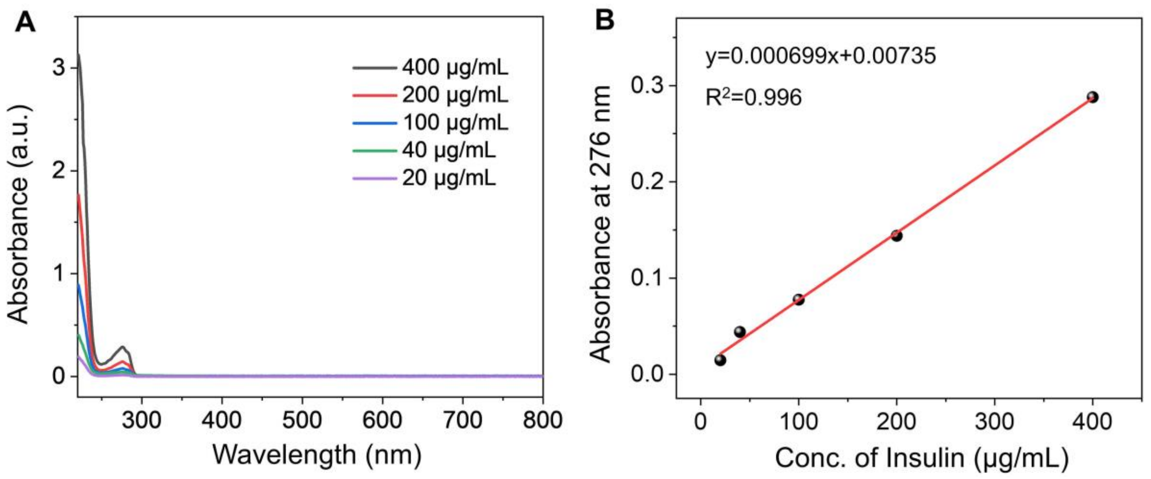

Figure S8. (A) UV-vis spectra recorded from aqueous solutions of insulin at concentrations of 20–400 μg mL^{-1} and (B) the corresponding calibration curve used for determining the concentrations of insulin released from the nanobottles.

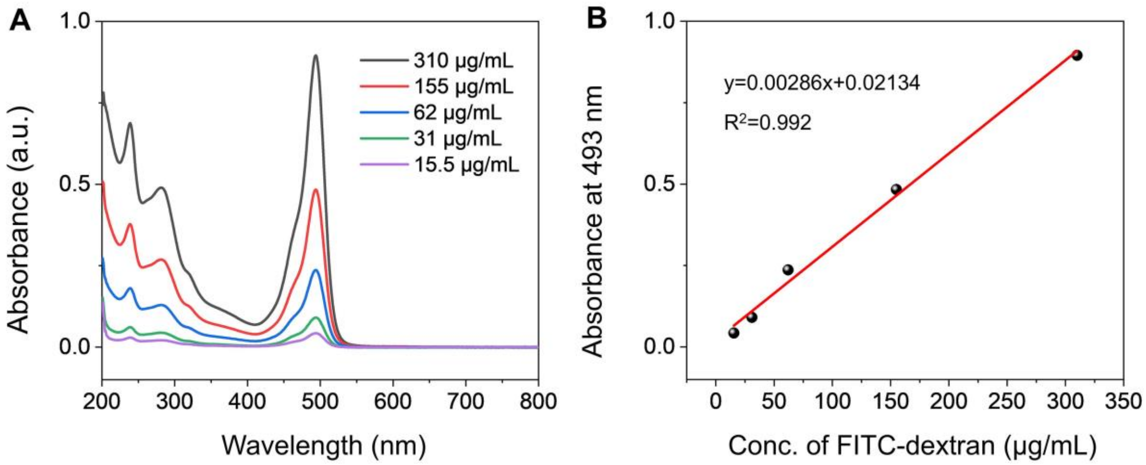

Figure S9. (A) UV-vis spectra of aqueous solutions of FITC-dextran at concentrations of 15.5–310 μg mL^{-1} and (B) the corresponding calibration curve used for determining the concentrations of FITC-dextran released from the nanobottles.

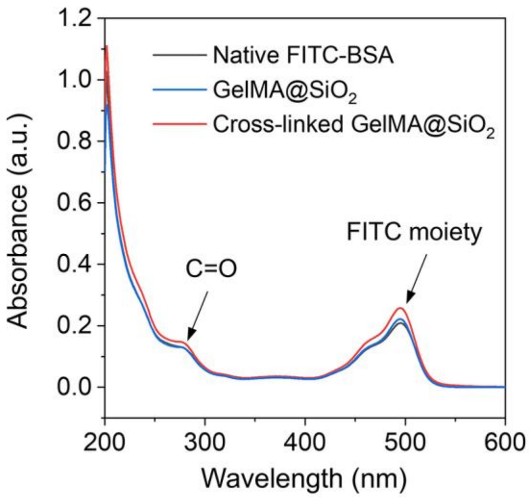

Figure S10. UV-vis spectra recorded from the aqueous solutions of native FITC-BSA and those released from the non-cross-linked and cross-linked GelMA@SiO_2 nanobottles.

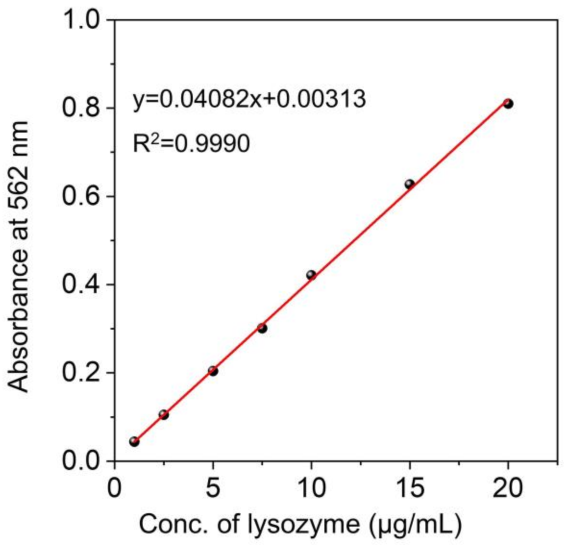

Figure S11. Calibration curve used for determining the concentration of lysozyme through the use of a Micro BCA Protein Assay Kit.

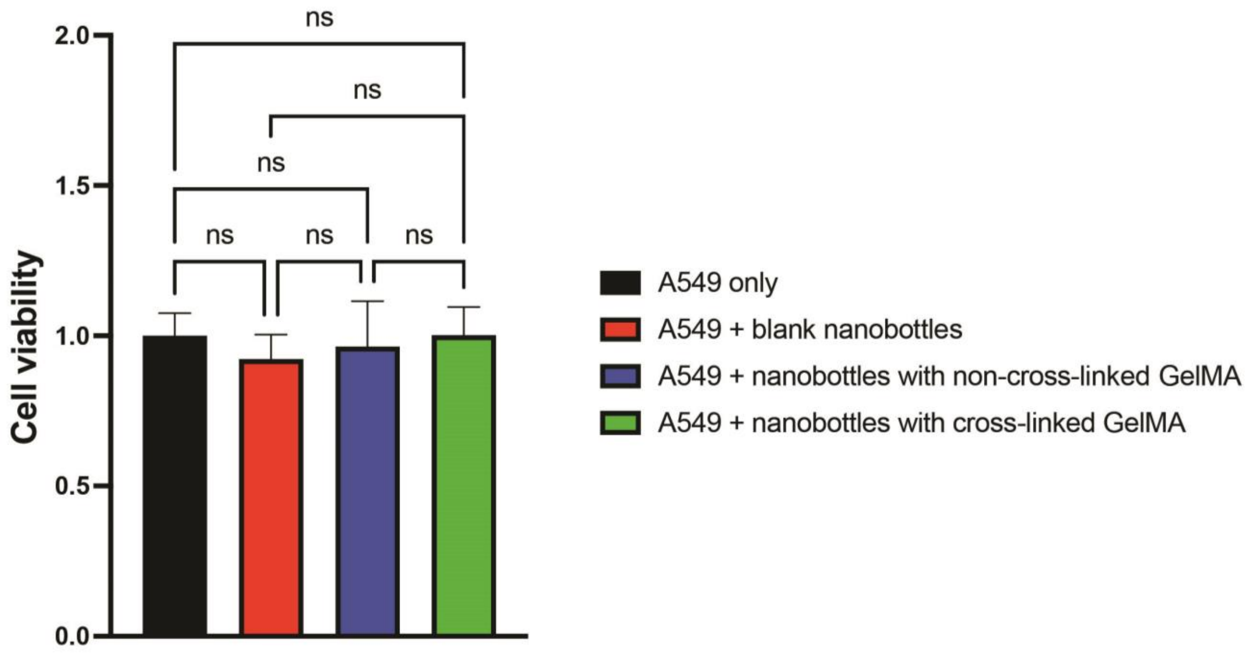

Figure S12. Cell viability of A549 cells after 3-day incubation with blank nanobottles, nanobottles loaded with non-cross-linked GelMA, and nanobottles loaded with cross-linked GelMA. No significant differences (ns) in viability were observed among the treatment groups compared to untreated cells, indicating low cytotoxicity of both nanobottle formulations and GelMA loadings. Data are presented as mean ± SD (n = 3).

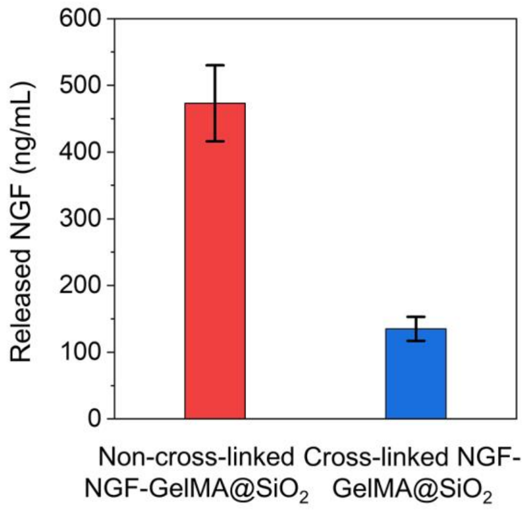

Figure S13. Concentrations of NGF released from the non- and cross-linked NGF-GelMA@SiO_2 nanobottles (*ca.* 4.0 mg), respectively, after incubation in medium at 37 °C for 3 days.

Table S1. Summary of the encapsulation efficiency (EE) of FITC-BSA, insulin, FITC-dextran, lysozyme, and NGF in the GelMA@SiO_2 nanobottles. W_0 and W_{drug} represent the weight of the drug in nanobottles and the weight of the drug added, respectively. EE is defined as W_0/W_{drug} (%).

Drug	Molecular weight (Da)	W_{drug} (μg)	W_0 (μg)	EE (%)
FITC-BSA	60,000	100	4.3	4.3
Insulin	5,300	400	30.1	7.5
FITC-dextran	4,000	200	6.3	3.2
Lysozyme	14,000	400	28.2	7.1
NGF	27,000	5	0.3	6.0

Table S2. Zeta potential measurements of SiO_2 nanobottles and representative payloads (insulin, FITC-dextran, and FITC-BSA) at physiological pH, illustrating differences in surface charge relevant to diffusion and release behavior.

	Zeta Potential (mV)
SiO_2 Nanobottles	-6.2 ± 0.95
Insulin	-17.23 ± 0.37
FITC-dextran	-5.99 ± 1.95
FITC-BSA	-9.39 ± 0.72

REFERENCES

(1) Qiu, J.; Chen, Z.; Chi, M.; Xia, Y. Swelling-Induced Symmetry Breaking: A Versatile Approach to the Scalable Production of Colloidal Particles with a Janus Structure. *Angew. Chem. Int. Ed.* **2021**, *60*, 12980–12984.
